# EARLY-LIFE PROTEIN TRANSLATION SPIKE DRIVES AGING VIA JUVENILE HORMONE/GERMLINE STEM CELL SIGNALING

**DOI:** 10.1093/geroni/igac059.520

**Published:** 2022-12-20

**Authors:** Harper Kim, Danitra Parker, Madison Hardiman, Erin Munkácsy, Steven Austad, Yi-dong Bai, James Mobley, Andrew Pickering

**Affiliations:** University of Alabama at Birmingham (UAB), Birmingham, Alabama, United States; University of Alabama at Birmingham (UAB), Birmingham, Alabama, United States; University of Alabama at Birmingham (UAB), Birmingham, Alabama, United States; University of Texas Health San Antonio, San Antonio, Texas, United States; University of Alabama at Birmingham, Birmingham, Alabama, United States; University of Texas Health San Antonio, San Antonio, Texas, United States; University of Alabama at Birmingham (UAB), Birmingham, Alabama, United States; University of Alabama at Birmingham, Hoover, Alabama, United States

## Abstract

Protein translation (PT) is high in early-adulthood across invertebrates, rodents, and humans but sharply declines thereafter. It has been implicitly assumed that elevated PT at young ages is beneficial to health and PT ends up dropping as a passive byproduct of aging. However, whether this holds true and how dynamic fluctuations in PT over time impact aging remain unknown. In Drosophila, we show that a transient PT spike in early-adulthood exerts long-lasting negative impacts on aging trajectories and proteostasis in later-life. Conversely, blocking the early-life PT spike robustly improves life-/health-span and prevents age-related protein aggregation. Further, greater early-life PT rise strongly predicts shorter future lifespan across fly strains and is observed in neurodegenerative disorders long before symptoms/pathologies appear. Proteomics-guided investigations revealed that during the early-adulthood PT rise, juvenile hormone triggers proteostatic dysfunction and drives aging via aggregation-prone large lipid transfer proteins. The early-life PT spike also transcriptionally represses stress responses essential for proteostasis maintenance and drives aging via germline stem cell signaling. Our findings suggest that PT is thereby suppressed after early-adulthood as an adaptive response to alleviate proteostatic burden, slow down aging, and optimize life-/health-span. We thus propose that the rise and fall in PT over time impact aging in the opposite direction from what was previously assumed. Our work provides a novel theoretical framework for understanding how lifetime PT dynamics regulate the onset of aging. Further, our study provides a foundation for future research, including whether high early-life PT spike is an early biological event driving neurodegeneration/age-related diseases.

